# Mass spectrometry data confirming tetrameric α-synuclein N-terminal acetylation

**DOI:** 10.1016/j.dib.2018.09.026

**Published:** 2018-09-14

**Authors:** Ricardo D. Fernández, Heather R. Lucas

**Affiliations:** Department of Chemistry, Virginia Commonwealth University, Richmond, VA, USA

## Abstract

Tetrameric α-synuclein (αS) is an elusive multimer of the dynamic neuronal protein implicated in Parkinson׳s disease. Through the data reported herein, we demonstrate that this high molecular weight multimer is N-acetylated. Coexpression of tetrameric αS in *Escherichia coli* with the NatB acetylase derived from yeast enables access to N-terminally acetylated αS (^NAc^αS), the native form in humans. Following purification and characterization as previously described by us in “Isolation of Recombinant Tetrameric N-acetylated α-synuclein” (Fernández and Lucas, 2018), the purified protein was excised from a native gel for confirmation of N-terminal acetylation. Through high-resolution mass spectrometry techniques, the identification of this helical tetramer as ^NAc^αS has been clearly demonstrated.

**Specifications table**

**Table:** 

Subject area	*Biochemistry*
More specific subject area	*Protein Mass Spectrometry and Post-translational Modification; Protein Multimer Isolation for Characterization*
Type of data	*Spectra, figures, tables*
How data was acquired	*Mass spectrometry (Thermo Scientific LTQ Orbitrap Velos and Waters Synapt G2Si); Gel electrophoresis (BN-PAGE)*
Data format	*Raw, analyzed, and graphical representation*
Experimental factors	*Protein co-expression and multimer isolation through ammonium sulfate precipitation, ion exchange chromatography, gel filtration, and gel electrophoresis*
Experimental features	*Exact mass of intact protein and CID fragmentation analysis of target peptides*
Data source location	*Virginia Commonwealth University, Richmond, VA, USA*
Data accessibility	*Data is within this article*
Related research article	*R.D. Fernández, H.R. Lucas, Isolation of Recombinant Tetrameric N-acetylated α-synuclein. Protein Expr. Purif. 152 (2018) 146–154*[1]

**Value of the data**

• The data demonstrates two complementary mass spectrometry techniques for verifying the N-terminal acetylation of recombinant human tetrameric α-synuclein.
• Utilization of BN-PAGE gel band excision can be used to successfully confirm exact mass protein identification through diffusion of intact protein.
• Fragmentation analysis following tryptic digestion of high molecular weight multimers preserves and verifies N-capping through *in cellulo* post-translational modification.
• The data solidifies that recombinant platforms can be employed to generate human proteins with their native N-terminal acetylation in *Escherichia coli*.

## 1 Data

**Fig. 1:**
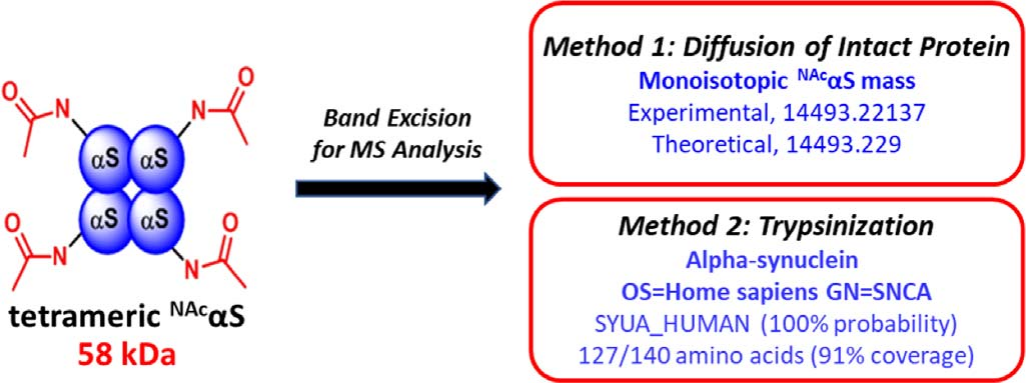
*Schematic representation of mass spectrometry techniques.* Following band excision from BN-PAGE (4–16%) gels, two methods were employed for characterization of the N-terminal peptide fragment of tetrameric ^NAc^αS: (1) diffusion of intact protein; and (2) trypsinization and peptide mapping.

**Fig. 2:**
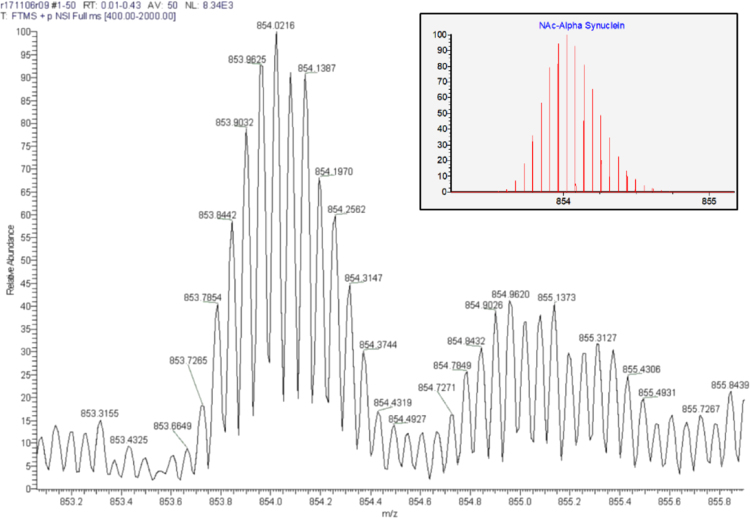
*Mass spectrum of the +17 charge state envelope of ^NAc^αS***.** Following excision of the tetrameric ^NAc^αS band from a BN-PAGE (4–16%) gel and diffusion of the protein into water, the exact mass for N-terminally acetylated αS was obtained (experimental monoisotopic mass, 14493.22137). The low abundance, higher molecular weight charge envelope that is also visible in this spectrum corresponds to nonspecific oxygenation of ^NAc^αS. *Inset*: Theoretical isotope distribution and relative abundances of [M+17H]^+17^ (^NAc^αS calculated monoisotopic mass, 14493.229).

**Fig. 3:**
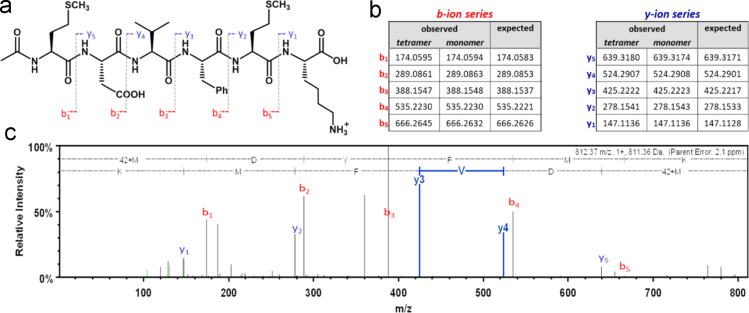
*N-terminal peptide mapping and ion fragmentation.* (A) Sketch of the N-terminal peptide fragment following trypsin digestion of ^NAc^αS, with overlaid b- and y-ion maps. (B) Tables with experimental and theoretical data for both the b-ion and y-ion series of tetrameric vs monomeric ^NAc^αS. (C) Annotated CID mass spectrum for tetrameric ^NAc^αS demonstrating sequence match; y_3_ and y_4_ are denoted to highlight the high mass accuracy (<1 ppm).

**Fig. 4:**
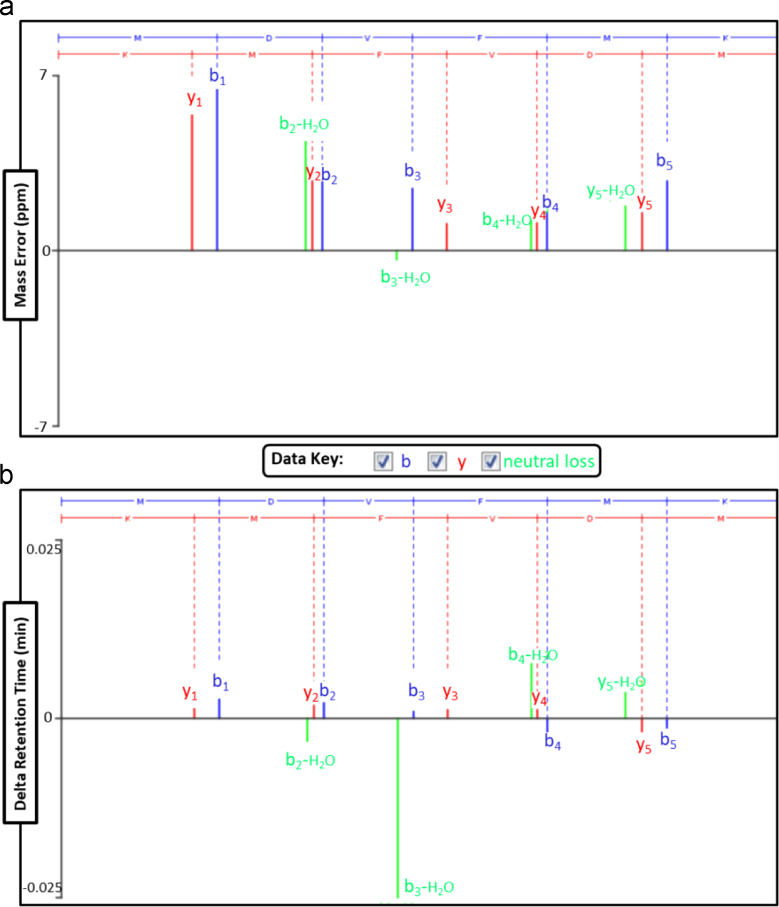
*Parametric analysis of CID fragmentation spectrum associated with the N-terminal fragment of tetrameric ^NAc^αS***.** (A) Mass error of all b/y-ion fragments are within +7 ppm. (B) Retention time of b/y-ion fragments varies by <0.01 min.

Fig. 1 shows a scheme that represents our strategy for mass spectrometry (MS) analysis. The cartoon signifies tetrameric ^NAc^αS and its theoretical molecular weight. After co-expression and purification, the purified protein was run on a blue native PAGE (BN-PAGE) gel. Excised gel bands were analyzed by high resolution mass spectrometry via two separate methods. Data collected through Method 1 (diffusion of intact protein) is shown in Fig. 2 for the dissociated noncovalent ^NAc^αS tetramer along with an inset of the theoretical isotopic distribution and relative abundance of an intact monomeric unit for comparison. Select data collected through Method 2 (trypsinization) is shown in Fig. 3 along with the associated peptide map of the N-terminal peptide as well as the theoretical b-/y-ion masses. Finally, Fig. 4 displays the quality of the mass data based on the low error and short retention times.

## 2 Experimental design, materials, and methods

### 2.1 Protein expression and purification

Tetrameric ^NAc^αS was expressed and purified as previously described [1], [2]. Verification of the multimeric conformer was confirmed by BN-PAGE, and the associated native gel for the intact protein has been described in the related research article [1]. N-terminal acetylation was verified by mass spectrometry following gel electrophoresis as described herein (see Fig. 1 for synopsis).

### 2.2 Diffusion of intact protein from excised BN-PAGE gel (Method 1)

The 58 kD gel band corresponding to tetrameric ^NAc^αS was excised from a BN-PAGE gel (Invitrogen) after destaining, and the purified protein was isolated through diffusion following band excision. The excised band was cut into small pieces and resuspended in ddH_2_O for 4 h [3]. Samples were then homogenized with a syringe, probe sonicated, and centrifuged at 14,500 g for 5 min. The supernatant was collected for MS analysis.

### 2.3 MS analysis of intact protein (Method 1)

The sample extracted from the 58 kD band of the tetrameric ^NAc^αS sample was analyzed by direct infusion onto a Thermo Electron LTQ Orbitrap Velos [1]. Exact mass determination for one unit of the homotetrameric ^NAc^αS was measured within less than 1 ppm of the theoretical mass (Fig. 2). Statistical analyses [4] of the isotopic distribution of the 17+ charge envelope verified the identification of N-terminally acetylated αS. Theoretical mass isotopic distributions and abundances were calculated using the IsoPro 3.1 simulator, which is based on the Yergey algorithm [5].

### 2.4 Band excision and sample processing for trypsin digestion (Method 2)

The 58 kD band was excised from a BN-PAGE gel of the tetrameric ^NAc^αS sample and divided into 1 mm segments to aid in digestion. Gel pieces were transferred to a siliconized tube and washed and destained in 200 µL 50% methanol overnight. The gel pieces were dehydrated in acetonitrile, rehydrated in 30 µL of 10 mM DTT in 0.1 M ammonium bicarbonate and reduced at room temperature for 0.5 h. The DTT solution was removed and the samples were alkylated in 30 µL 50 mM iodoacetamide in 0.1 M ammonium bicarbonate at room temperature for 0.5 h. The reagent was removed and the gel pieces were dehydrated in 100 µL acetonitrile. The acetonitrile was removed and the gel pieces were rehydrated in 100 µL 0.1 M ammonium bicarbonate. The pieces were dehydrated in 100 µL acetonitrile, the acetonitrile removed and the pieces were completely dried by vacuum centrifugation. The gel pieces were rehydrated in 20 ng/µL trypsin in 50 mM ammonium bicarbonate on ice for 10 min. Any excess trypsin solution was removed and 20 µL 50 mM ammonium bicarbonate added. The samples were digested overnight at 37 °C, and the peptides formed from the digestion were extracted from the polyacrylamide in two 30 µL aliquots of 50% acetonitrile/5% formic acid. These extracts were combined and evaporated to 15 µL for MS analysis. This protocol was also followed for the monomeric ^NAc^αS gel band.

### 2.5 MS analysis of digested protein (Method 2)

Samples were analyzed by a Waters Synapt G2Si mass spectrometer system with a nanospray ion source interfaced to a Waters M-Class C18 reversed-phase capillary column. The peptides were injected onto the trap and analytical columns, and the peptides were eluted from the column by an acetonitrile/0.1% formic acid gradient at a flow rate of 0.4 µL/min over 60 min. The nanospray ion source was operated at 3.5 kV. A lockspray compound was used to improve the mass accuracy of the analysis. The digests were analyzed using the double play capability of the instrument acquiring full scan mass spectra at low collision energy to determine peptide molecular weights and product ion spectra at high collision energy to determine amino acid sequence. This mode of analysis produces approximately 10,000 collision-induced dissociation (CID) spectra of ions ranging in abundance over several orders of magnitude. Not all CID spectra are derived from peptides. The data were analyzed by database searching using the ProteinLynx Global Server (PLGS, Waters Corp.) search algorithm against Uniprot׳s Human database. Fragmentation data was prepared for publication using Scaffold Q+ (Figs. 3 and 4). Theoretical b-/y-ion masses were determined using the ProteinProspector v.5.22.1, MS-Product, data mining program (Fig. 3B).
