# Supplementary material for: Mass spectrometry data confirming tetrameric α-synuclein N-terminal acetylation
Source: Data Brief. 2018 Sep 14;20:1686–91. doi: 10.1016/j.dib.2018.09.026 (PMC6157607; doi:10.1016/j.dib.2018.09.026)
Supplement: Supplementary file 1 — Supplementary material [file mmc1.pdf]

VIRGINIA COMMONWEALTH UNIVERSITY

VCU

Make it real.

College of Humanities and Sciences
Department of Chemistry

Oliver Hall, Kapp Wing
1001 W. Main St.
Richmond, Virginia 23284-2006

RE: CONFLICT OF INTEREST STATEMENT

September 6, 2018

Dear DIB Managing Editor,

With regards to our **DIB-D-18-01888R1** manuscript entitled "*Mass Spectrometry Data Confirming Tetrameric a-Synuclein N-terminal Acetylation*" submitted to *Data in Brief*, we have provided a statement below confirming that there are no conflicts of interest. Although mentioned in the EES hub, there was not a linked form.

There are no known conflicts of interest associated with this publication and there has been no significant financial support for this work that could have influenced its outcome. The manuscript has been read and approved by all authors and the regulations of our institution concerning intellectual property have been followed.

Sincerely,

Heather R. Lucas, Ph.D.
Assistant Professor of Chemistry Biology, Division of Inorganic Chemistry
Department of Chemistry, Virginia Commonwealth University
hrlucas@vcu.edu
804-828-7512
